# Prevalence of family history of cancer in the NC-CCAPH consortium of Japan

**DOI:** 10.1038/s41598-023-30048-6

**Published:** 2023-02-22

**Authors:** Sarah Krull Abe, Hikaru Ihira, Tetsuji Minami, Takuya Imatoh, Yosuke Inoue, Kota Tsutsumimoto, Nozomu Kobayashi, Rena Kashima, Maki Konishi, Takehiko Doi, Masayuki Teramoto, Isamu Kabe, Sangyoon Lee, Makoto Watanabe, Seitaro Dohi, Yukie Sakai, Yukiko Nishita, Naho Morisaki, Hisateru Tachimori, Yoshihiro Kokubo, Taiki Yamaji, Hiroyuki Shimada, Tetsuya Mizoue, Norie Sawada, Shoichiro Tsugane, Motoki Iwasaki, Manami Inoue

**Affiliations:** 1 Division of Prevention, National Cancer Center Institute for Cancer Control, 5-1-1 Tsukiji, Chuo-Ku, Tokyo, 104-0045 Japan; 2 Division of Cohort Research, National Cancer Center Institute for Cancer Control, Tokyo, Japan; 3 Department of Cancer Epidemiology, Division of Social Medicine, Graduate School of Medicine, The University of Tokyo, Tokyo, Japan; 4 Department of Epidemiology and Prevention, Center for Clinical Sciences, Research Institute, National Center for Global Health and Medicine, Tokyo, Japan; 5 Department of Preventive Gerontology, Center for Gerontology and Social Science National Center for Geriatrics and Gerontology, Aichi, Japan; 6 Cancer Screening Center, National Cancer Center Hospital, Tokyo, Japan; 7 Division of Screening Technology, National Cancer Center Institute for Cancer Control, Tokyo, Japan; 8 Department of Preventive Cardiology, National Cerebral and Cardiovascular Center, Osaka, Japan; 9 KUBOTA Corporation, Tokyo, Japan; 10 Division of Preventive Healthcare, National Cerebral and Cardiovascular Center, Osaka, Japan; 11 Mitsui Chemicals, Inc., Tokyo, Japan; 12 Department of Epidemiology of Aging, Center for Gerontology and Social Science, National Center for Geriatrics and Gerontology, Aichi, Japan; 13 Department of Social Medicine, National Center for Child Health and Development, Tokyo, Japan; 14 Department of Clinical Data Science, Clinical Research & Education Promotion Division, National Center of Neurology and Psychiatry, Tokyo, Japan; 15 Endowed Course for Health System Innovation, Keio University School of Medicine, Tokyo, Japan; 16 Division of Epidemiology, National Cancer Center Institute for Cancer Control, Tokyo, Japan; 17 National Institute of Health and Nutrition, National Institutes of Biomedical Innovation, Health and Nutrition, Tokyo, Japan

**Keywords:** Cancer, Cancer prevention

## Abstract

The objective of this study was to identify the prevalence of family history of cancer using cohorts participating in the Japanese National Center Cohort Collaborative for Advancing Population Health (NC-CCAPH). We pooled data from seven eligible cohorts of the Collaborative with available data on family history of cancer. Prevalence of family history of cancer and corresponding 95% confidence intervals are presented for all cancers and selected site-specific cancers for the total population and stratified by sex, age, and birth cohort. Prevalence of family history of cancer increased with age ranging from 10.51% in the 15 to 39 year age category to 47.11% in 70-year-olds. Overall prevalence increased in birth cohorts from ≤ 1929 until 1960 and decreased for the next two decades. Gastric cancer (11.97%) was the most common site recorded for family members, followed by colorectal and lung (5.75%), prostate (4.37%), breast (3.43%) and liver (3.05%) cancer. Women consistently had a higher prevalence of family history of cancer (34.32%) versus men (28.75%). Almost one in three participants had a family history of cancer in this Japanese consortium study highlighting the importance of early and targeted cancer screening services.

## Introduction

Family history of cancer is defined as having one or more first-degree relatives (FDR) (e.g., parent, sibling or child) diagnosed with cancer^1,2^. Individuals with a family history of breast, ovarian, uterine, and colorectal cancer, in particular, are known to be at increased risk of getting such cancers^1,2^. This risk increases with younger age at diagnosis of the relative^1,3^. Multiple FDR with a history of cancer affects an individual’s cancer risk^1,3^. While the complex interaction of genetic and environmental exposures contributing to the association between family history of cancer and cancer risk have been extensively studied^4–6^, exact pathways remain inconclusive^7^.

The US Preventive Task Force updated guidelines in 2020 for site-specific cancers such as colorectal, lung, breast, pancreas, cervix and ovary cancer^8^. Given that early detection is possible through effective screening methods, the U.S. guidelines highlight available screening programs for individuals with a family history^1,8^. In addition, clinical services also focused on prevention such as BRCA gene mutation testing or breast chemoprevention^9^. In the UK risk-reducing mastectomies are offered to women who have been identified at high risk^10^.

In Japan, the National Cancer Center (NCC) Japan publishes and updates cancer screening guidelines^11–15^. However, the recommendations do not specify physician assessment of family history. Family history is not always assessed and assessment methods are inconsistent though the rationale for obtaining this history is well established^4^. Several epidemiological studies exist on family history of cancer and various cancer outcomes in Japan^4^. However, a broad overview and update on the current prevalence of family history of total and site-specific cancer stratified by sex for age and birth cohorts are lacking. For example, no Japanese studies were included in the 2019 paper on trends by the Hereditary Breast Cancer Study Group^10^.

The objective of this study was to identify the prevalence of family history of cancer using cohorts participating in the Japanese National Center Cohort Collaborative for Advancing Population Health (NC-CCAPH).

## Materials and methods

### Study population

The Japan Health Research Promotion Bureau (JH Bureau) was launched in April 2020, to create innovation in research and development (R&D) and medical care by coordinating six national centers for advanced and specialized medicine in Japan (6NC)^16^. The six national centers are: NCC, the National Cerebral and Cardiovascular Center (NCVC), the National Center of Neurology and Psychiatry, the National Center for Global Health and Medicine (NCGM), the National Center for Child Health and Development, and the National Center for Geriatrics and Gerontology (NCGG). Additional details can be found on the JH bureau homepage^16^.

Study-specific data was pooled from seven of nine eligible independent cohorts (Supplementary file) participating in the National Center Cohort Collaborative for Advancing Population Health (NC-CCAPH) or 6NC for short. Four cohorts of the National Cancer Center contributed data: Japan Public Health Center Study I (JPHC I)^17^, Japan Public Health Center Study II (JPHC II)^17^, Japan Public Health Center NEXT Study (JPHC-NEXT)^18^, and the National Cancer Center Japan-Screening Cohort (JaSCo) Study^19^. The Japan Epidemiology Collaboration on Occupational Health Study (J-ECOH)^20^ of the NCGM joined and the National Center for Geriatrics and Gerontology-Study of Geriatric Syndromes (NCGG-SGS)^21^ and the Suita Study (Suita) of NCVC^22^. Participants in the JPHC II Study Osaka Suita Public Health Center area were excluded (n = 10,960) to avoid potential overlap with participants in the Suita Study. None of the other study areas overlapped, thus eliminating the possibility of duplication. The remaining two eligible cohorts participating in the consortium could not be included as they lacked data on family history of cancer. One of the cohorts would have added a younger birth cohort. This study was approved by the Institutional Review Board of the National Cancer Center Japan (approval number 2018–194). All methods were carried out in accordance with relevant guidelines and regulations. Informed consent was obtained from all subjects.

### Analysis

Family history was assessed at baseline using questionnaires. While other questions such as the food frequency questionnaires (FFQ) have been validated, the family history questions have not been verified. A respondent was considered to have a family history of cancer if at least one family member (parent or sibling) had cancer^7^. We included respondents with complete information for age, birth year and who responded to relevant family history exposure questions. Individuals were counted once for total cancer even if they had multiple site-specific cancers. Prevalence estimates and respective 95% confidence intervals (CIs) were calculated by pooling the data and presented for total family history of cancer, parent and sibling separately, by sex and site-specific cancers for all cohorts except J-ECOH, for which only total cancer was available. Estimates were calculated using “ci proportion” in Stata to obtain the percent. Prevalence was presented in 10 year age categories from 40 to > 70 and birth cohorts < 1929 to 1979. All analyses were performed using Stata Statistical Software: Release 17, College Station, Texas.

## Results

Over 343,000 participants with available data on family history of cancer and age from seven Japanese cohort studies were included in the study. Survey years ranged from 1989 to 2020 and age at baseline ranged from 15 to 104 (Table 1). Birth year ranged from 1906 to 2004 (Table 1). The total prevalence of family history of cancer varied between cohorts from 18.82% (J-ECOH) to 63.05% (JaSCo) (Table 1). J-ECOH represents a relatively young workers cohort whereas JPHC-NEXT, JaSCo, NCGG-SGS and Suita include older participants (Table 1).

**Table 1:** Overview of participating cohorts of the Japanese Six National Centers (6NC) consortium.

Cohort study name	Center	Participants, number	Survey years	Age, range	Birth year, range	% of FH of cancer
Japan Public Health Center Study I (JPHC I)	NCC	50,245	1990	40–59	1931–1950	20.22
Japan Public Health Center Study II (JPHC II)	NCC	52,256	1993–1994	40–69	1923–1952	21.96
Japan Public Health Center NEXT Study (JPHC-NEXT)	NCC	114,054	2011–2016	40–75	1936–1976	41.56
Japan-Screening Cohort (JaSCo)	NCC	14,673	2004–2013	40–79	1925–1965	63.05
Japan Epidemiology Collaboration on Occupational Health (J-ECOH)	NCGM	84,085	2012–2020	15–59	1927–2004	18.82
National Center for Geriatrics and Gerontology-Study of Geriatric Syndromes (SGS)	NCGG	19,388	2019–2020	60–104	1915–1960	54.43
Suita Study (Suita)	NCVC	8,352	1989–2003	30–79	1906–1967	29.68
Total		343,053	1989–2020	15–104	1906–2004	35.67

National Cancer Center (NCC), National Cancer for Global Health and Medicine (NCGM), and National Center for Geriatrics and Gerontology (NCGG), National Cerebral and Cardiovascular Center (NCVC); Age refers to age at baseline.

Prevalence of a family history of cancer increased with age ranging from 10.51% (95% CI 10.22to 10.88) in the 15 to 39 year age category to 47.11% (95% CI 46.59 to 47.63) in 70-year-olds (Table 2). Overall prevalence increased in birth cohorts from ≤ 1929 until 1960 and decreased for the next two decades (Table 3). Gastric cancer (11.97%; 95% CI 11.84 to 12.10) was the most common site recorded for family members, followed by colorectal and lung (5.75%; 95% CI 5.66 to 5.84), prostate (4.37%; 95% CI 4.26 to 4.47), breast (3.43%; 95% CI 3.36 to 3.50) and liver (3.05%; 95% CI 2.99 to 3.12) cancer. Women consistently had a higher prevalence of family history of cancer (34.32%; 95% CI 34.08 to 34.56) versus men (28.75%; 95% CI 28.55 to 28.96) (Tables 2 and 3).

**Table 2:** Prevelance of family history of cancer and corresponding 95% confidence intervals by age in seven Japanese cohort studies.

Age	Participants	Total cancer		Parent		Sibling		Breast		Colorectal		Gastric		Liver		Lung		Prostate	
40 to 49 years	92,573	25.71	(25.42–25.99)	23.86	(23.55–24.18)	3.57	(3.44–3.71)	2.24	(2.13–2.35)	3.39	(3.26–3.53)	7.47	(7.28–7.67)	1.90	(1.80–2.00)	3.21	(3.08–3.34)	3.86	(3.64–4.10)
Men	50,944	24.69	(24.32–25.08)	23.20	(22.75–23.66)	3.13	(2.95–3.33)	2.04	(1.89–2.10)	3.25	(3.06–3.45)	7.21	(6.93–7.49)	1.75	(1.61–1.90)	3.07	(2.89–3.27)	3.64	3.32–3.98)
Women	41,629	26.94	(26.52–27.37)	24.46	(24.03–24.90)	3.98	(3.78–4.18)	2.42	(2.26–2.58)	3.52	(3.33–3.72)	7.71	(7.44–7.99)	2.04	(1.89–2.19)	3.32	(3.14–3.51)	4.06	(3.74–4.39)
50 to 59 years	96,185	33.24	(32.94–33.54)	29.2	(28.88–29.51)	8.03	(7.85–8.22)	2.74	(2.63–2.86)	4.78	(4.64–4.94)	10.91	(10.69–11.13)	2.75	(2.64–2.86)	5.10	(4.94–5.25)	4.58	(4.36–4.80)
Men	50,588	32.22	(31.81–32.63)	28.82	(28.37–29.28)	7.11	(6.86–7.38)	2.46	(2.30–2.62)	4.67	(4.46–4.89)	10.64	(10.33–10.96)	2.62	(2.46–2.79)	4.93	(4.71–5.15)	4.50	(4.20–4.82)
Women	45,597	34.38	(33.94–34.81)	29.52	(29.09–29.96)	8.85	(8.58–9.12)	3.00	(2.83–3.16)	4.89	(4.68–5.10)	11.15	(10.85–11.45)	2.86	(2.70–3.02)	5.24	(5.03–5.46)	4.64	(4.35–4.95)
60 to 69 years	75,415	39.63	(39.28–39.98)	31.03	(30.69–31.37)	15.91	(15.64–16.19)	3.94	(3.79–4.09)	7.17	(6.98–7.37)	14.32	(14.05–14.58)	3.59	(3.45–3.73)	7.06	(6.87–7.25)	4.06	(3.89–4.24)
Men	37,913	38.74	(38.25–39.23)	31.42	(30.93–31.92)	14.64	(14.27–15.03)	3.48	(3.28–3.68)	7.12	(6.84–7.41)	14.14	(13.77–14.52)	3.50	(3.30–3.70)	7.00	(6.75–7.30)	4.11	3.86–4.36)
Women	37,502	40.53	(40.03–41.02)	30.67	(30.19–31.14)	17.08	(16.69–17.47)	4.36	(4.15–4.58)	7.21	(6.94–7.49)	14.48	(14.11–14.85)	3.68	(3.48–3.88)	7.09	(6.82–7.37)	4.02	(3.78–4.26)
≥ 70 years	36,047	47.11	(46.59–47.63)	27.57	(27.10–28.04)	24.22	(23.78–24.67)	6.44	(6.18–6.71)	10.01	(9.69–10.33)	19.02	(18.60–19.44)	5.06	(4.83–5.30)	9.89	(9.58–10.22)	5.01	(4.78–5.25)
Men	17,412	45.08	(44.34–45.82)	28.16	(27.48–28.84)	22.47	(21.84–23.10)	6.34	(5.97–6.73)	9.03	(8.59–9.48)	18.15	(17.56–18.76)	4.53	(4.21–4.86)	9.24	(8.79–9.70)	4.14	(3.83–4.46)
Women	18,635	49.01	(48.29–49.73)	27.03	(26.39–27.67)	25.83	(25.20–26.47)	6.53	(6.17–6.91)	10.89	(10.44–11.36)	19.80	(19.22–20.40)	5.55	(5.22–5.90)	10.48	(10.04–10.94)	5.80	(5.46–6.15)
Total	343,053	31.23	(31.08–31.38)	27.95	(27.78–28.12)	11.23	(11.00–11.24)	3.43	(3.36–3.50)	5.75	(5.66–5.84)	11.97	(11.84–12.10)	3.05	(2.99–3.12)	5.75	(5.66–5.84)	4.37	(4.26–4.47)
Men	190,399	28.75	(28.55–28.96)	27.85	(27.60–28.11)	10.16	(10.00–10.33)	3.15	(3.05–3.25)	5.53	(5.40–5.66)	11.66	(11.48–11.84)	2.88	(2.78–2.97)	5.57	(5.44–5.70)	4.13	(3.98–4.28)
Women	152,654	34.32	(34.08–34.56)	28.04	(27.80–28.28)	11.99	(11.82–12.17)	3.68	(3.58–3.78)	5.94	(5.82–6.07)	12.26	(12.08–12.44)	3.21	(3.12–3.31)	5.75	(5.66–5.84)	4.59	(4.44–4.74)

Participants for family history of total cancer; total includes those < 40 years and missing age; J-ECOH only total cancer; Suita Cohort only total, parent and sibling; Age 15–39 from J-ECOH and Suita: 42,833 participants 10.51% (95% CI: 10.22–10.80).

**Table 3:** Prevalance of family history of cancer and corresponding 95% confidence intervals by birth cohort in seven Japanese cohort studies.

Birth cohort	Participants	Total cancer		Parent		Sibling		Breast		Colorectal		Gastric		Liver		Lung		Prostate	
≤ 1929	15,653	23.64	(22.97–24.31)	14.23	(13.69–14.79)	12.30	(11.79–12.83)	1.10	(0.93–1.13)	1.80	(1.58–2.05)	6.08	(5.67–6.51)	1.39	(1.20–1.61)	1.83	(1.61–2.08)	5.66	(4.23–7.34)
Men	7,229	23.52	(22.54–24.51)	14.62	(13.82–15.46)	11.84	(11.11–12.61)	1.06	(0.81–1.34)	1.93	(1.58–2.32)	6.16	(5.55–6.81)	1.18	(0.92–1.50)	1.79	(1.47–2.17)	3.04	(1.63–5.15)
Women	8,424	23.74	(22.84–24.67)	13.89	(13.16–14.65)	12.69	(11.99–13.42)	1.14	(0.90–1.42)	1.69	(1.41–2.02)	6.01	(5.47–6.59)	1.57	(1.29–1.89)	1.87	(1.56–2.21)	7.86	(5.67–10.55)
1930–1939	53,451	28.50	(28.12–28.88)	19.39	(19.05–19.73)	12.09	(11.82–12.37)	2.23	(2.10–2.36)	3.34	(3.19–3.50)	9.80	(9.54–10.06)	2.45	(2.32–2.59)	3.95	(3.78–4.12)	5.18	(4.78–5.60)
Men	25,718	28.42	(27.87–28.97)	20.16	(19.66–20.66)	11.60	(11.22–12.01)	2.30	(2.12–2.50)	3.31	(3.09–3.54)	9.69	(9.33–10.07)	2.36	(2.17–2.56)	3.94	(3.70–4.19)	4.17	(3.67–4.71)
Women	27,733	28.57	(28.04–29.11)	18.68	(18.22–19.14)	12.54	(12.15–12.94)	2.16	(1.99–2.34)	3.37	(3.15–3.59)	9.90	(9.54–10.26)	2.55	(2.36–2.74)	3.95	(3.72–4.20)	6.22	(0.56–0.69)
1940–1949	98,286	35.61	(35.31–35.91)	26.73	(26.45–27.01)	13.87	(13.66–14.10)	3.64	(3.52–3.76)	5.91	(5.76–6.07)	13.13	(12.91–13.35)	3.24	(3.13–3.35)	6.11	(5.96–6.27)	4.25	(4.08–4.43)
Men	48,253	34.80	(34.38–35.23)	27.18	(26.78–27.60)	12.63	(12.33–12.94)	3.24	(3.08–3.41)	5.63	(5.42–5.85)	12.81	(12.51–12.13)	3.00	(2.84–3.16)	5.91	(5.70–6.14)	4.09	(3.85–4.35)
Women	50,033	36.38	(35.96–36.81)	26.31	(25.93–26.71)	15.02	(14.71–15.34)	4.01	(3.83–4.18)	6.18	(5.97–6.40)	13.42	(13.12–13.73)	3.46	(3.30–3.63)	6.29	(6.08–6.51)	4.40	(4.16–4.66)
1950–1959	67,902	40.47	(40.10–40.84)	37.12	(36.71–37.52)	10.23	(9.98–10.49)	4.27	(4.10–4.45)	7.83	(7.60–8.06)	14.57	(14.28–14.87)	3.71	(3.56–3.88)	7.92	(7.70–8.16)	4.41	(4.22–4.60)
Men	36,705	38.12	(37.62–38.62)	36.25	(35.67–36.93)	8.73	(8.39–9.07)	3.80	(3.57–4.04)	7.56	(7.23–7.89)	14.00	(13.58–14.44)	3.53	(3.31–3.77)	7.61	(7.28–7.94)	4.23	(3.96–4.51)
Women	31,197	43.23	(42.68–43.78)	37.90	(36.34–38.46)	11.58	(11.21–11.95)	4.70	(4.45–4.95)	8.08	(7.76–8.40)	15.09	(14.67–15.51)	3.88	(3.65–4.11)	8.21	(7.89–8.53)	4.56	(4.30–4.84)
1960–1969	48,539	34.63	(34.21–35.06)	37.59	(37.03–38.14)	4.65	(4.44–4.90)	4.10	(3.87–4.33)	7.57	(7.27–7.88)	11.48	(11.11–11.85)	3.38	(3.17–3.59)	6.26	(5.98–6.54)	4.57	(4.33–4.82)
Men	29,560	31.65	(31.12–32.18)	36.12	(35.31–36.93)	3.87	(3.55–4.21)	3.78	(3.46–4.12)	7.13	(6.70–7.58)	10.82	(10.30–11.36)	3.24	(2.94–3.55)	5.93	(5.54–6.35)	4.57	(4.23–4.94)
Women	18,979	39.29	(38.60–39.99)	38.87	(38.10–38.46)	5.33	(4.98–5.70)	4.38	(4.06–4.71)	7.95	(7.53–8.39)	12.06	(11.55–12.58)	3.50	(3.21–3.80)	6.54	(6.15–6.94)	4.57	(4.25–4.91)
1970–1979	31,140	22.03	(21.57–22.50)	28.16	(27.32–29.01)	2.69	(2.30–3.13)	4.03	(3.67–4.41)	5.24	(4.83–5.67)	7.76	(7.27–8.28)	2.13	(1.87–2.42)	3.73	(3.38–4.10)	3.24	(2.92–3.59)
Men	20,628	19.62	(19.08–20.17)	26.40	(25.19–27.63)	2.51	(2.10–2.98)	3.83	(3.32–4.39)	5.20	(4.61–5.85)	7.69	(6.98–8.46)	1.96	(1.60–2.38)	3.49	(3.01–4.03)	2.73	(2.30–3.21)
Women	10,512	26.76	(25.92–27.62)	29.66	(28.50–30.84)	2.69	(2.30–3.13)	4.20	(3.70–4.74)	5.27	(4.71–5.86)	7.82	(7.16–8.53)	2.27	(1.91–2.68)	3.93	(3.45–4.45)	3.68	(3.22–4.19)
Total	343,053	31.23	(31.08–31.38)	27.95	(27.78–28.12)	11.23	(11.00–11.24)	3.43	(3.36–3.50)	5.75	(5.66–5.84)	11.97	(11.84–12.10)	3.05	(2.99–3.12)	5.75	(5.66–5.84)	4.37	(4.26–4.47)
Men	190,399	28.75	(28.55–28.96)	27.85	(27.60–28.11)	10.16	(10.00–10.33)	3.15	(3.05–3.25)	5.53	(5.40–5.66)	11.66	(11.48–11.84)	2.88	(2.78–2.97)	5.57	(5.44–5.70)	4.13	(3.98–4.28)
Women	152,654	34.32	(34.08–34.56)	28.04	(27.80–28.28)	11.99	(11.82–12.17)	3.68	(3.58–3.78)	5.94	(5.82–6.07)	12.26	(12.08–12.44)	3.21	(3.12–3.31)	5.75	(5.66–5.84)	4.59	(4.44–4.74)

Participants for family history of total cancer; total includes those birth year is > 1980; J-ECOH only total cancer; Suita only total, parent and sibling; > 1980 only total cancer J-ECOH participants 28,082; prevalence 7.33 (95%CI: 7.03–7.64).

## Discussion

One in three participants had a family history of cancer in this Japanese study, which was similar to the study finding reported in the United States in 2006^7^. A consistently higher prevalence of family history of cancer among women versus men was also in line with the study conducted in the United States^7^. Gastric cancer was the most common family history of cancer site in this Japanese study, followed by lung, colorectal, prostate, breast and liver cancer. In comparison, the US-based study by Ramsey et al. found breast, followed by lung, colorectal, and prostate cancer, to be the most common cancer sites of those assessed. Even though breast cancer rates are lower in Japan, the incidence is increasing^23^. The US population is ethnically heterogeneous compared to a homogenous population in Japan. The proportion of some cancers which have strong genetic factors could differ by ethnicity^24^. In contrast to our study, a Swedish Cancer Registry study found a higher 20.15% proportion of family history of prostate cancer versus 4.37% in this Japanese study^25^.

A meta-analysis of case–control studies in Eastern and Central Europe found family history of lung cancer to be a risk factor for lung cancer, particularly in younger subjects under age 50^5^. The prevalence of family history of lung cancer among Japanese increased by age group from 3.21% in participants in their 40 s to 9.89% in the ≥ 70 year olds. The prevalence was similar among men and women at 5.75% for total family history of cancer all age groups. In a large-scale population-based study in Utah, USA, 30% of individuals had a family history in at least one FDR, second-degree relative, or third-degree relative, however this percent decreased dramatically when only considering FDRs^26^.

In Japan, the prevalence of infection-related cancers such as a family history of gastric and liver cancer, 11.87% and 3.03%, was higher than those reported Europe and North America. In fact, the prevalence of gastric cancer family history was surprisingly high even though the incidence has been on a decreasing trend. Infection risk may be linked with birth cohorts. Specifically for *Helicobacter pylori* (*H. pylori*) the evidence is mixed whether the infection is the cause of gastric cancer^27^. General improvements in hygiene have contributed to the decline of the *H.pylori* infection in the Japanese population^27^ as well as scaling up targeted gastric cancer screening programs may contribute. At the political level in Japan, prevention of infection and detection of carriers, and antiviral treatment have contributed to a substantial reduction of liver cancer incidence.

The reasons why the proportion of people with a family history of cancer is increasing are complex. These trends are observed in other countries. Common reasons may include expanded longevity leading to more cancer cases and improved cancer detection rates due to the spread of screening and development of testing techniques^28^. These factors combined may complicate the interpretation of family history for each birth cohort. Family history in our study increased by birth cohort until 1970, participants born after this were younger at baseline with younger parents who may have not yet been diagnosed. On the other hand, it can be speculated that parents of participants in the earlier cohorts did not notice they had cancer or died before the occurrence of cancer because of shorter life expectancy. Additionally, an interaction between life expectancy, lifestyle such as diet and genetics must be considered^29,30^. Genetics within the Japanese population remained stable during the study period. The high percentage of affected persons highlights the importance of early and targeted cancer screening services.

The government develops guidelines for organized cancer screening and societies/associations create screening guidelines for opportunistic screening. However, some of the descriptions may not obtain consensus from all stakeholders. For example, professional medical societies such as the Japan Association of Breast Cancer Screening^31^, Japanese Association for Cancer Detection and Diagnosis, Japanese Breast Cancer Society (JBCS)^32^, The Japan Lung Cancer Society^33^, Japan Cancer Society^34^, Japanese Society for Cancer of the Colon and Rectum, and The Japan Urological Association^35^ publish their own site-specific statements. Among them, a family history of cancer is included only for opportunistic screening for prostate cancer.

The main strength of this study is that the data comes from several large-scale population-based cohorts in Japan, making the study representative. Age at baseline spanned almost 100 years (15–104) and birth cohorts cover nearly a century (1915–2004). This study may help predict the burden of screening on physicians, clinics, insurers, and systems. The self-reported information from the cohorts should be sufficiently reliable. While the family history questions were not validated, other parts of the questionnaires such as FFQ indicated relatively high quality. In the JaSCo study for example, consistency of responses to all items was inspected by research assistants in the presence of study subjects. Despite these strengths, several limitations warrant mentioning. First, two cohorts did not collect cancer site-specific data. Parent versus sibling family history was not differentiated in all cohorts. This information was presented separately for the whole family history of cancer. Second, this analysis did not consider additional details such as age of relative at diagnosis and socioeconomic factors. These disparities are smaller in Japan than in Europe and the US and thus likely contributed less to an individual’s risk^36^. Third, false reporting or misdiagnosis, especially in the earlier birth cohorts, cannot be ruled out. Cancer death may be equal to cancer incidence, leading to competing risk of dying from stroke instead of stomach cancer. Also, participants, especially in the early birth cohorts, may know of cancer death of a family member but not necessarily specifically which cancer site, which may result in a further under-estimation in the site proportion of family history of site-specific cancer. Memory may be ambiguous and people do not know accurate information about their parents’ and sibling. In the Twenty-First Century individuals may access and use digitalized personal health information and add their family history at any time. This information can be useful as a public health tool. Forth, the data of family history depended on self-report.

Albeit the limitations as mentioned above, this Japanese National Center Cohort Collaborative for Advancing Population Health study, including nearly 343,000 participants, provides important information on family history of cancer prevalence. Definition of “high risk” family history in cancer screening and prevention guidelines should be reviewed. Future research should adopt risk prediction models for Japan’s family history and site-specific cancers. Additionally, including multi-generational biobank data would help improve the accuracy and validity of family history of cancer and applying findings to clinical and prevention strategies.

## Supplementary Information

Supplementary Information.

## Data Availability

The data underlying this manuscript cannot be shared publicly due to the privacy of study participants. A collaboration with each participating cohort study is required to access the data. Requests can be made to the NC-CCAPH Consortium Secretariat 6NCC@ncc.go.jp.

## Abbreviations

FDR: First-degree relative
*H. pylori*: *Helicobacter pylori*
FFQ: Food Frequency Questionnaires
JPHC I: Japan Public Health Center Study I
JPHC II): Japan Public Health Center Study II
JPHC-NEXT: Japan Public Health Center NEXT Study
JSCCR: Japanese Society for Cancer of the Colon and Rectum
JUA: The Japan Urological Association
J-ECOH: The Japan Epidemiology Collaboration on Occupational Health
MHLW: Ministry of Health Labor and Welfare
NC-CCAPH or 6NC: National Center Cohort Collaborative for Advancing Population Health
NC: National Cancer Center
JaSCo Study: National Cancer Center Japan-Screening Cohort
NCGM: National Center for Global Health and Medicine
NCVC: National Cerebral and Cardiovascular Center
NCGG): National Center for Geriatrics and Gerontology
NCGG-SGS: National Center for Geriatrics and Gerontology-Study of Geriatric Syndromes
Suita: The Suita Study
