# Supplementary material for: Prevalence of family history of cancer in the NC-CCAPH consortium of Japan
Source: Sci Rep. 2023 Feb 22;13:3128. doi: 10.1038/s41598-023-30048-6 (PMC9947111; doi:10.1038/s41598-023-30048-6)
Supplement: Supplementary file 1 [file 41598_2023_30048_MOESM1_ESM.docx]

Supplementary file 1. Profiles of participating cohorts

Cohort	Study subjects	Survey method	Reference
**National Cancer Center**			
JPHC I	A population-based cohort. Data were collected from five public health center areas across Japan.	Self-administered questionnaire	J Epidemiol. 2001;11(6 Suppl):S3-7.
JPHC II	A population-based cohort. Data were collected from six public health center areas across Japan.	Self-administered questionnaire	J Epidemiol. 2001;11(6 Suppl):S3-7.
JPHC-NEXT	A population-based cohort. Data were collected from in 16 municipalities of seven prefectures across Japan.	Self-administered questionnaire	J Epidemiol. 2020 Jan 5;30(1):46-54.
JaSCo	A hospital-based cohort. Data were collected from examinees of Cancer Screening Center, National Cancer Center Hospital Japan.	Self-administered questionnaire	Jpn J Clin Oncol 2006;36(5)301–308.
**National Cancer for Global Health and Medicine**			
J-ECOH*	A company-based cohort. Data were collected during annual health check-ups organized by each participating worksite.	Self-administered questionnaire	http://epid.ncgm.go.jp/research/images/res-009.pdf
**National Center for Geriatrics and Gerontology**			
NCGG-SGS	A community-based cohort in Aichi in Japan.	Self-administered questionnaire	J. Clin. Med. 2019; 8(10): 1554
**National Cerebral and Cardiovascular Center**			
Suita	An urban cohort based on random sampling of Japanese residents of Suita, Osaka in Japan	Physician or nurse-administered questionnaires	Hypertension.2008;52(4):652-659

Abbreviations: Japan Public Health Center-based prospective Study (JPHC), Japan Public Health Center NEXT Study (JPHC-NEXT); National Cancer Center Japan-Screening Cohort (JaSCo) Study, Japan Epidemiology Collaboration on Occupational Health (J-ECOH), Study of Geriatric Syndromes (SGS), and the Suita Study (Suita)

* Given that questions asked in the annual health check-ups were not under the control of the J-ECOH study group, some worksites did not collect information on family history of cancer. The information presented here is based on the data collected in worksites where the information was available.
